# Mesenchymal Stromal Cells Inhibit Neutrophil Effector Functions in a Murine Model of Ocular Inflammation

**DOI:** 10.1167/iovs.17-23067

**Published:** 2018-03

**Authors:** Sharad K. Mittal, Alireza Mashaghi, Afsaneh Amouzegar, Mingshun Li, William Foulsham, Srikant K. Sahu, Sunil K. Chauhan

**Affiliations:** 1 Schepens Eye Research Institute, Massachusetts Eye and Ear, Harvard Medical School, Boston, Massachusetts, United States; 2 Department of Ophthalmology, Beijing Hospital, National Center of Gerontology, Dong Dan, Beijing, People's Republic of China; 3 L.V. Prasad Eye Institute, Bhubaneswar, Odisha, India

**Keywords:** mesenchymal stromal cells, neutrophils, cornea, ocular, inflammation

## Abstract

**Purpose:**

Neutrophil-secreted effector molecules are one of the primary causes of tissue damage during corneal inflammation. In the present study, we have investigated the effect of stromal cells in regulating neutrophil expression of tissue-damaging enzymes, myeloperoxidase (MPO), and N-elastase (ELANE).

**Methods:**

Bone marrow–purified nonhematopoietic mesenchymal stromal cells and formyl-methionyl-leucyl-phenylalanine–activated neutrophils were cocultured in the presence or absence of Transwell inserts for 1 hour. Neutrophil effector molecules, MPO and ELANE, were quantified using ELISA. In mice, corneal injury was created by mechanical removal of the corneal epithelium and anterior stroma approximating one third of total corneal thickness, and mesenchymal stromal cells were then intravenously injected 1 hour post injury. Corneas were harvested to evaluate MPO expression and infiltration of CD11b^+^Ly6G^+^ neutrophils.

**Results:**

Activated neutrophils cocultured with mesenchymal stromal cells showed a significant 2-fold decrease in secretion of MPO and ELANE compared to neutrophils activated alone (*P* < 0.05). This suppressive effect was cell–cell contact dependent, as stromal cells cocultured with neutrophils in the presence of Transwell failed to suppress the secretion of neutrophil effector molecules. Following corneal injury, stromal cell–treated mice showed a significant 40% decrease in MPO expression by neutrophils and lower neutrophil frequencies compared to untreated injured controls (*P* < 0.05). Reduced MPO expression by neutrophils was also accompanied by normalization of corneal tissue structure following stromal cell treatment.

**Conclusions:**

Mesenchymal stromal cells inhibit neutrophil effector functions via direct cell–cell contact interaction during inflammation. The current findings could have implications for the treatment of inflammatory ocular disorders caused by excessive neutrophil activation.

Following ocular injury, innate immune responses lead to deleterious inflammatory tissue damage, primarily through the recruitment of activated neutrophils. Despite their role as an essential arm of innate immunity, activated neutrophils contribute greatly to the collateral tissue damage associated with acute injury and inflammation.^1^ Effector molecules released by activated neutrophils, including the enzymes myeloperoxidase (MPO) and N-elastase (ELANE), have been implicated in inflammatory damage of the cornea.^2–4^ Current nonspecific anti-inflammatory treatments (such as corticosteroids) are associated with serious side effects, including infection, cataract, and glaucoma.^5,6^ Thus, there is a pressing need for the development of novel therapeutic strategies that can curb tissue damage by selectively inhibiting pathogenic neutrophil pathways.

Mesenchymal stromal cells are tissue-resident cells that have been shown to regulate the function of various innate immune cells (including neutrophils, natural killer cells, and mast cells) in a wide array of inflammatory disorders.^7,8^ Stromal cells have been reported to suppress neutrophil apoptosis, which leads to the increased survival of neutrophils at the site of injury.^9^ Moreover, studies have shown that stromal cells inhibit neutrophil infiltration of inflamed tissues, in part through the secretion of TNF-α-stimulated gene 6 protein (TSG-6).^10^ Previous work from our group has shown that in vitro–expanded stromal cells suppress ocular inflammation following corneal injury.^11,12^ Although the propensity of stromal cells to modulate neutrophil survival and migration has been reported, the regulation of neutrophil effector functions by stromal cells at the ocular surface has not been fully investigated.

In the present study, we investigated the effect of stromal cells in regulating the secretion of neutrophil effector molecules, including proteases and cytokines, which cause tissue damage. Using both an in vivo murine model of ocular inflammaton and in vitro coculture assays, we demonstrate that mesenchymal stromal cells limit ocular tissue damage by inhibiting the release of MPO and ELANE by neutrophils in a cell–cell contact-dependent manner.

## Methods

### Animals

Six- to 8-week-old C57BL/6NCrl wild-type mice (Charles River Laboratories, Wilmington, MA, USA) were used in these experiments. Given that previous studies have shown similar corneal inflammation in male and female mice,^13–15^ we used male mice to maintain homogeneity in this study. The protocol was approved by the Schepens Eye Research Institute Animal Care and Use Committee, and all animals were treated according to the ARVO Statement for the Use of Animals in Ophthalmic and Vision Research.

### Ocular Injury Model

Mice were deeply anesthetized, and corneal injury was created as previously described.^12,16^ Briefly, the central cornea was marked by a 2-mm trephine, and using the tip of a handheld motor brush (AlgerBrush II; Alger Company, Inc., Lago Vista, TX, USA), the corneal epithelium and anterior stroma were removed mechanically (approximately one third of total corneal thickness). Upon completion of the procedure, triple antibiotic ointment (neomycin and polymyxin B sulfates and bacitracin zinc ophthalmic ointment USP; Bausch + Lomb, Wilmington, MA, USA) was applied to the injured eyes, and a subcutaneous injection of buprenorphine was given to mice to minimize injury-induced pain. To study the in vivo effects of stromal cells, mice were randomly divided into saline-treated control and stromal cell–treated group (*n* = 5–6 mice/group). In vitro expanded and characterized stromal cells (0.5 × 10^6^ cells/100 μL sterile saline) were injected into the tail veins of mice at 1 hour post injury. Mice were euthanized at two separate time points following injury; corneas were harvested at 24 hours post injury to examine neutrophil function, and eyeballs were harvested at 48 hours post injury to evaluate corneal thickness.

### Corneal Tissue Digestion

Single-cell suspensions were prepared from corneas as previously described.^11^ In brief, corneas were digested in RPMI media (Lonza, Walkersville, MD, USA) containing 2 mg/mL collagenase type IV (Sigma-Aldrich Corp., St. Louis, MO, USA) and 2 mg/mL DNase I (Roche, Basel, Switzerland) for 45 minutes at 37°C and then filtered through a 70-μm cell strainer.

### Cell Culture Assays

Due to the cornea harboring very low numbers of stromal cells and neutrophils, these cells were isolated from bone marrow for our in vitro experiments. Neutrophils were isolated from bone marrow of C57BL/6 mice using a neutrophil isolation kit (purity ≥ 95%) (MACS; Miltenyi Biotec, Inc., San Diego, CA, USA).^17,18^ Purified neutrophils were cultured alone or stimulated with fMLP (formyl-methionyl-leucyl-phenylalanine, 1 μM; Sigma-Aldrich Corp.) for 1 hour.^19,20^ Bone marrow–derived mesenchymal stromal cells (stromal cells) were generated by culturing bone marrow cells using the plastic adherence method and characterized as described previously.^11,12^ Stromal cells were passaged every 3 to 5 days and were used for experiments at passage three. Stromal cells were stimulated with IL-1β (100 ng/mL; Biolegend, San Diego, CA, USA) for 24 hours.^12^ For coculture assays, neutrophils were cultured alone or on stromal cell monolayer at the ratio of 1:1 for 1 hour. For TSG-6 neutralization experiments, cocultures were pretreated with a standard maximal concentration (10 μg/mL) of anti-TSG-6 antibody (AF2326; R&D Systems, Minneapolis, MN, USA) for 1 hour and were then stimulated with fMLP for an additional 1 hour. Two mice were used in each experiment, and each experiment was repeated three times.

### Transwell Experiments

To perform the Transwell coculture assays, Transwell inserts with polycarbonate membrane (0.4-μm pore size; Corning, NY, USA) were used to prevent neutrophil–stromal cell contact in 24-well plates. Neutrophils stimulated with fMLP were placed in the lower chambers, and stromal cells were cultured in the upper chambers with a 1:1 stromal cell-to-neutrophil ratio. After 1 hour, supernatants were collected for the analysis of MPO and ELANE secretion using ELISA described below (*n* = 3 well/group, and repeated three times in three independent experiments).

### Enzyme-Linked Immunosorbent Assay

Levels of MPO and ELANE in culture supernatants from neutrophil and stromal cell coculture assays were analyzed using commercially available murine ELISA kits (R&D Systems; Abcam, Cambridge, MA, USA) per the manufacturer's instructions.

### Flow Cytometry

Single-cell suspensions were prepared and stained with fluorochrome-conjugated monoclonal antibodies against CD11b, Ly6G for their cell surface expression, and MPO for intracellular expression of neutrophils. Appropriate isotype controls were used. Antibodies against CD45, CD34, and CD29 were used for the phenotypic characterization of stromal cells. For cell survival assays, neutrophils were stained with propidium iodide (PI). Stained cells were analyzed using a flow cytometer (LSR II; BD Biosciences, San Jose, CA, USA) and FlowJo software (FlowJo LLC, Ashland, OR, USA). All antibodies and isotypes controls were purchased from Biolegend.

### Real-Time PCR

Total RNA was isolated using a kit (RNeasy Micro Kit; Qiagen, Valencia, CA, USA) and reverse transcribed into cDNA using reverse transcriptase (Superscript III; Invitrogen, Carlsbad, CA, USA). Quantitative real-time PCR was then performed using preformulated Taqman-based probes for murine *Mpo* (Mm01298424-m1), *Elane* (Mm00469310_m1), *Il-1b* (Mm00434228_m1), and glyceraldehype-3-phosphate dehydrogenase (*Gapdh*, Mm99999915_gl) (Taqman Universal PCR Mastermix; Thermo Fisher Scientific, Walthan, MA, USA) in a Mastercycler Realplex2 (Eppendorf, Hamburg, Germany). The results were analyzed by the comparative threshold cycle method and normalized to *Gapdh* as an internal control.^11,12^

### Histology

Whole eyeballs were harvested after 48 hours of injury. Paraformaldehyde-fixed cross sections were stained with hematoxylin and eosin (H&E) and examined for corneal tissue structures using a bright-field microscope (Nikon Eclipse E800; Nikon Instruments, Melville, NY, USA) at 20× magnification.^11^

### Statistical Analysis

A Mann-Whitney *U* test was performed to determine significance, which was set at *P* ≤ 0.05. Data are presented as the mean ± standard deviation (SD). Results shown are representative of three independent experiments. Sample sizes were estimated on the basis of previous experimental studies on corneal injury and inflammation^12,21,22^ and using Harvard-MGH Biostatistics Center software (available free of charge at http://hedwig.mgh.harvard.edu/sample_size/js/js_parallel_quant.html).

## Results

### Corneal Injury Promotes the Infiltration of Neutrophils at the Ocular Surface

**Figure 1:**
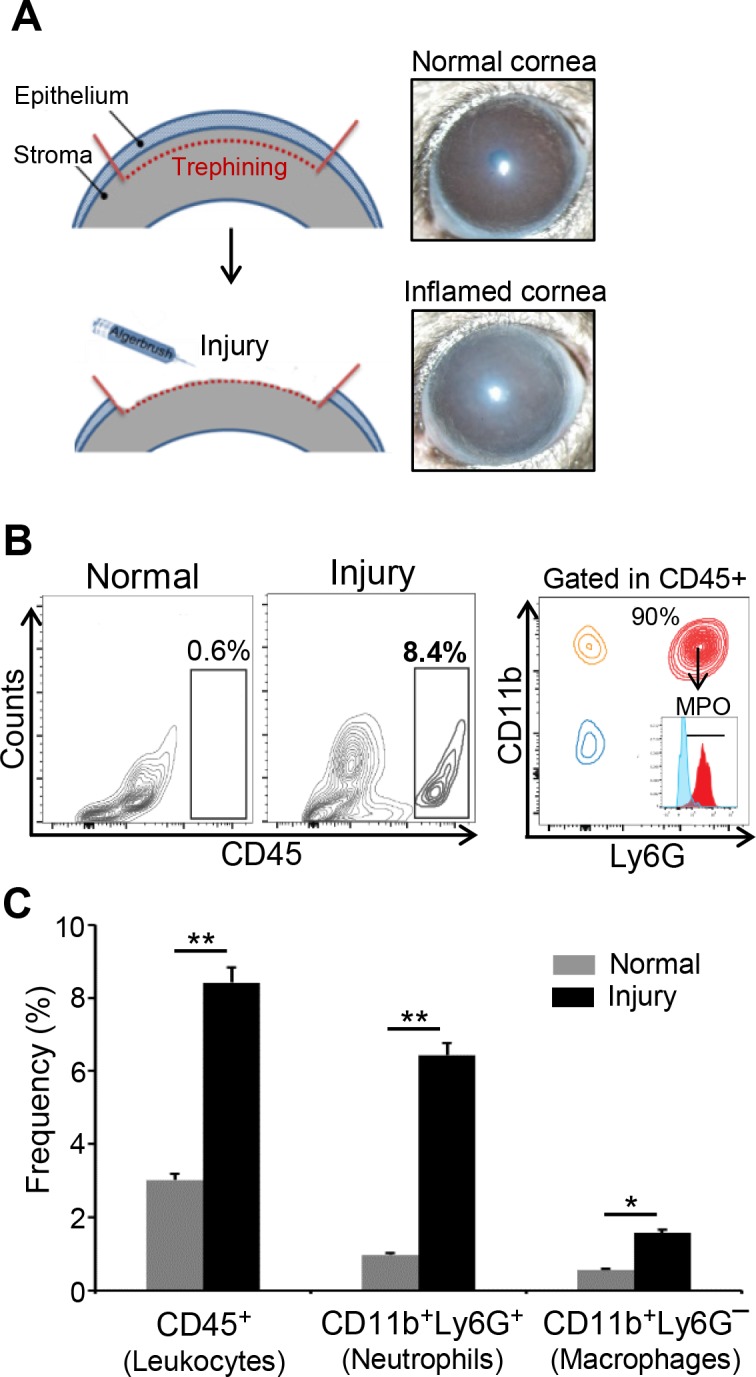
Corneal injury promotes the infiltration of neutrophils at the ocular surface. (A) Schematic diagrams and representative images showing the mouse model of corneal inflammation induced by mechanical removal of corneal epithelium and anterior stroma using Alger brush-II. Corneas were harvested 24 hours after injury induction. (B) Single-cell suspensions were prepared, and flow cytometry was performed to determine the frequencies of total CD45^+^ inflammatory cells and CD11b^+^Ly6G^+^MPO^+^ cells (neutrophils) within CD45^+^ cell population in naive and injured mice. (C) Bar diagram showing the frequencies of CD45^+^ cells, CD11b^+^Ly6G^+^ (neutrophils) and CD11b^+^LyG^-^ (macrophages) within CD45^+^ cell in the corneas of naive and injured mice. Representative data from three independent experiments are shown, and each experiment consisted of five animals. Data are represented as mean ± SD. *P < 0.05; **P < 0.01.

For this study, we utilized a well-established murine model of corneal inflammation.^3,12^ Low vascularity and paucity of resident immune cells in the cornea make this model an excellent in vivo system in which to study the recruitment and function of immune cells in corneal inflammation. We first investigated the infiltration of inflammatory cells to the cornea after injury. Corneal injury was created by mechanical removal of corneal epithelium and anterior stroma (Fig. 1A). Noninjured corneas served as control. Corneas were harvested 24 hours post injury for further analysis. Flow-cytometry analysis of corneal cells revealed increased frequencies of CD45^+^ inflammatory cells at the ocular surface compared to control noninjured corneas (Figs. 1B, 1C). Our results further showed that the majority of CD45^+^ cells were CD11b^+^Ly6G^+^MPO^+^ neutrophils (Figs. 1B, 1C). There was also a moderate increase in the frequencies of CD11b^+^Ly6G^−^ cells (macrophages) in injured corneas compared to noninjured controls (Fig. 1C).

### Stromal Cells Suppress the Neutrophil Effector Functions Without Inducing Cell Death

**Figure 2:**
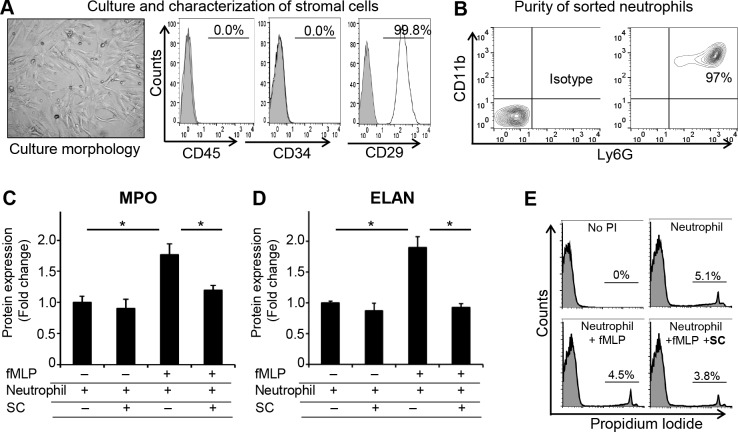
Stromal cells suppress the neutrophil effector functions without inducing cell death. (A) Stromal cells (SCs) were isolated from bone marrow and expanded in vitro using a plastic adherence method. Micrographs showing the morphology of stromal cells in culture at passage two. Characterization of in vitro–expanded stromal cells using flow cytometry confirmed their phenotype as CD45^−^CD34^−^CD29^+^ cells. (B) Flow-cytometry plots showing the purity of CD11b^+^Ly6G^+^ neutrophils isolated from bone marrow using magnetic activated cell sorting. (C, D) Neutrophils were stimulated with fMLP (1 μM) in the presence or absence of stromal cells for 1 hour. ELISA was performed to evaluate (C) MPO and (D) ELANE secretion in culture supernatants. (E) Representative flow-cytometry histograms showing the frequencies of PI-positive neutrophils in different groups. Representative data from three independent experiments are shown, and data are represented as mean ± SD. *P < 0.05.

Next, we investigated the effect of stromal cells on neutrophil effector functions. To this aim, neutrophils were cocultured with stromal cells, and the secretion of MPO and ELANE by neutrophils was assessed using ELISA. Given that the cornea harbors very low numbers of stromal cells and neutrophils, we isolated these cells from bone marrow for our in vitro experiments. Characterization of in vitro–expanded stromal cells using flow cytometry revealed these cells to be positive for the stromal cell marker CD29 and negative for the hematopoietic cell markers CD45 and CD34 (Fig. 2A). Additionally, neutrophils were isolated from the bone marrow using magnetic activated cell sorting (purity ≥ 95%) (Fig. 2B). Neutrophils were stimulated with fMLP, a neutrophil stimulant that is produced by necrotic cells during sterile inflammation, and cultured with or without stromal cells for 1 hour. ELISA analysis of culture supernatants revealed that fMLP treatment significantly enhanced the secretion of MPO (1609 ± 159 pg/mL) and ELANE (722 ± 66 pg/mL) by neutrophils. It is interesting that the secretion of these effector molecules was dramatically suppressed in fMLP-stimulated neutrophils cultured with stromal cells (MPO: 1090 ± 67; ELANE: 353 ± 21 pg/mL) compared to fMLP-stimulated neutrophils cultured alone (Figs. 2C, 2D). Next, we investigated whether stromal cells suppress neutrophil effector functions by promoting cell death, using PI viability staining. No significant difference in the frequencies of PI-positive neutrophils (dead cells) between stromal cell–treated (3.81 ± 0.79) and untreated (4.59 ± 1.2) cultures (Fig. 2E) was observed, further indicating that stromal cell–mediated suppression of neutrophil function is not due to induction of cell death.

### Stromal Cells Inhibit Neutrophil Function in a Contact-Dependent and TSG-6-Independent Manner

**Figure 3:**
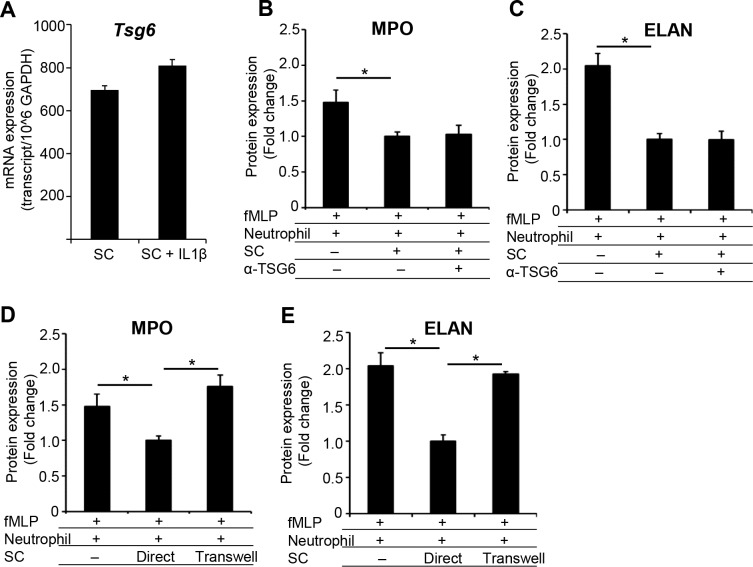
Stromal cells suppress neutrophil function in a contact-dependent and TSG-6-independent manner. (A) Stromal cells were cultured in medium alone or in medium stimulated with inflammatory cytokine IL-1β (100 ng/mL) for 24 hours. Tsg-6 mRNA expression (normalized to internal control GAPDH) was evaluated using real-time PCR. (B, C) fMLP-activated neutrophil and stromal cell cocultures were pretreated with TSG-6–neutralizing antibody or control isotype antibody. Culture supernatants were collected to evaluate the secretion of (B) MPO and (C) ELANE using ELISA. (D, E) Transwell coculture assay was performed with fMLP-stimulated neutrophils in the lower chamber and stromal cells in the upper chamber for 1 hour at 1:1 ratio. (D) MPO and (E) ELANE secretion in culture supernatants were measured using ELISA. Results are representative of three independent experiments. The values shown represent mean ± SD, *P < 0.05.

Next, we set to delineate the mechanism by which stromal cells suppress neutrophil functions. Mesenchymal stromal cells regulate the immune response through the secretion of paracrine inhibitory factors as well as by cell–cell interactions via membrane-bound receptor molecules.^23^ Indeed, the soluble factor TSG-6, which is primarily produced by human^21^ and mouse^24^ mesenchymal stromal cells, has been shown to suppress neutrophil infiltration of the injured cornea.^21^ Our observation that stromal cells express *Tsg-6* mRNA in the steady state as well as in inflammatory conditions (Fig. 3A) led us to investigate whether stromal cell–mediated suppression of neutrophil function is TSG-6 dependent. To determine this, fMLP-stimulated neutrophils and stromal cells were cocultured in the presence of TSG-6-neutralizing antibody for 1 hour. ELISA analysis of culture supernatants demonstrated that TSG-6 neutralization did not alter the secretion of MPO (1330 ± 163 pg/mL) or ELANE (320 ± 48 pg/mL) by fMLP-stimulated neutrophils cultured with stromal cells, compared to neutrophil–stromal cell cocultures without TSG-6 neutralization (MPO: 1299 ± 79; ELANE: 331 ± 28 pg/mL) (Figs. 3B, 3C). These results suggest that stromal cell–mediated suppression of neutrophil effector functions is independent of TSG-6 secretion. To determine whether direct cell–cell interactions contribute to the inhibitory effect of stromal cells on release of these effector molecules, fMLP-stimulated neutrophils were cultured with stromal cells with or without Transwell inserts. ELISA analysis of culture supernatants demonstrated that unlike direct cocultures, stromal cells cultured in Transwells failed to suppress the secretion of MPO (2274 ± 209 pg/mL) and ELANE (638 ± 12.6 pg/mL) by neutrophils (Figs. 3D, 3E).

### Stromal Cells Suppress Neutrophil Effector Functions and Tissue Damage During Ocular Inflammation

**Figure 4:**
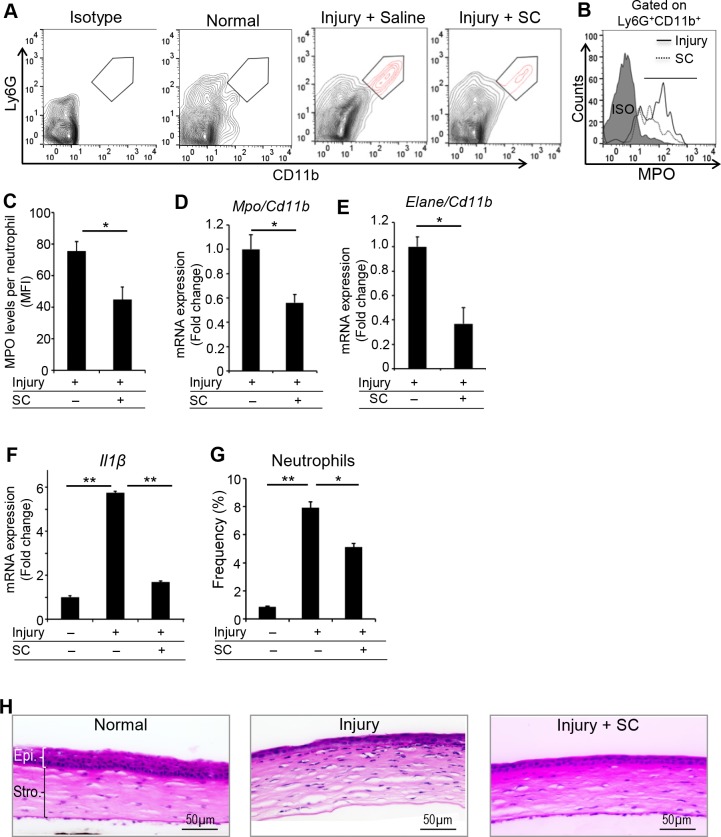
Stromal cells suppress the neutrophil function after corneal injury. Stromal cells were intravenously administered 1 hour post corneal injury in C57BL/6 mice. Healthy mice without injury and saline-treated injured mice served as controls. Corneas were harvested after 24 hours. Corneal single-cell suspensions were prepared, and flow cytometry was performed. (A) Representative flow cytometry plots showing CD11b^+^Ly6G^+^ cells (neutrophils; red) at ocular surface of indicated mice groups. (B, C) Histogram and bar diagram showing the expression (mean fluorescent intensity) of MPO by CD11b^+^Ly6G^+^ cells. (D) Mpo and (E) Elane mRNA expression within ocular surface infiltrating neutrophils (normalized to first GAPDH and then to CD11b transcripts) was quantitated using real-time PCR. (F) Expression of IL-1β inflammatory cytokine (normalized to GAPDH) at the ocular surface was evaluated using real-time PCR. (G) Bar diagram showing the frequencies of neutrophils at ocular surface of indicated mice groups. (H) Cross sections (×20) of corneas harvested at 48 hours post injury in different treatment groups were stained with H&E to visualize inflammatory cells and corneal tissue structures. Results are representative of two independent experiments. Each group consisted of five to six animals in each experiment. The values shown represent mean ± SD, *P < 0.05; **P < 0.01.

Finally, using our in vivo model of injury-induced corneal inflammation, we determined whether stromal cells could regulate the neutrophil effector functions in the inflamed cornea. We have shown previously that bone marrow–derived stromal cells home specifically to the injured cornea.^22,25^ Here, we intravenously injected in vitro–expanded stromal cells to injured mice at 1 hour following injury and harvested corneas after 24 hours. Saline-treated injured mice and mice without injury served as controls. Flow-cytometry analysis of corneal cells revealed that stromal cell–treated mice showed reduced levels of MPO (2-fold decrease) in the infiltrated CD11b^+^ Ly6G^+^ neutrophils compared to the control group (Figs. 4A, 4B, 4C). We confirmed the stromal cell–mediated suppression of MPO and ELANE expression by neutrophils at the mRNA level, with an approximate 2.5-fold decrease in *Mpo* and *Elane* mRNA observed in mice treated with stromal cells relative to the control group (Figs. 4D, 4E). We also evaluated the ocular surface expression of the inflammatory cytokine IL-1β, which is expressed at higher levels in activated neutrophils.^26^ Real-time PCR analysis showed significantly reduced expression of IL-1β in the stromal cell–treated group compared to untreated injured mice (Fig. 4F). Consistent with previous reports,^3,16^ we found reduced frequencies of neutrophils in stromal cell–treated mice compared to control groups (Fig. 4G). Given the central role of neutrophil-derived MPO and elastase in tissue damage during inflammation, we evaluated corneal tissue architecture after injury using H&E staining (Fig. 4H). Histopathologic analysis of injured corneas harvested at 48 hours post injury demonstrated restoration of normal corneal tissue structures, including stromal thickness and reduced inflammatory cell infiltration in stromal cell–treated mice compared to untreated injured mice. Collectively, these findings indicate that stromal cells suppress the neutrophil effector functions and subsequent tissue damage after corneal injury.

## Discussion

Dysregulated neutrophil activation leads to persistent inflammation and subsequent tissue damage. In this study, we investigated the effect of stromal cells in regulating neutrophil effector functions during eye inflammation. Using a murine model of ocular injury, we report that stromal cells inhibit the secretion of the tissue-degrading enzymes MPO and ELANE by neutrophils and limit ocular inflammation. Moreover, we demonstrate that the observed stromal cell–mediated suppression of neutrophil function is primarily dependent on direct cell–cell interactions and is independent of stromal cell–secreted TSG-6.

The role of certain immune cells in curbing the inflammatory response has been established in a wide range of immune disorders.^27,28^ For example, regulatory T cells are crucial for modulating the antigen-specific immune response,^28^ and myeloid-derived suppresser cells and M2 macrophages are involved in regulating non–antigen-specific innate inflammation of nonocular tissues such as the liver, kidneys, and lungs.^29,30^ Our study reveals that stromal cells, a type of nonimmune cell, are also critical for regulating nonspecific inflammation through their suppression of neutrophil effector functions.

Mesenchymal stromal cells inhibit neutrophil apoptosis and promote their survival through secretion of IL-6.^9^ However, our study provides novel evidence that stromal cells also regulate neutrophil secretion of the tissue-damaging molecules MPO and ELANE without promoting neutrophil cell death. We thus decided to further delineate the mechanisms of stromal cell suppression of neutrophil function. Previous studies have shown that stromal cell–derived TSG-6 interacts with CXCL8 and suppresses the infiltration of neutrophils in inflammatory conditions such as acute pancreatitis.^10,31^ At the eye, TSG-6 has been shown to attenuate the recruitment of neutrophils to the cornea after chemical and mechanical injuries.^3^ It is interesting that neutralization of TSG-6 in our stromal cell–neutrophil coculture assays did not abrogate the suppressive effects of stromal cells on MPO and ELANE secretion by neutrophils. Stromal cells have been reported to regulate the function of myeloid cells by direct cell-to-cell contact and by secreting soluble factors such as prostaglandin E2, indoleamine 2,3-dioxygenase transforming growth factor-β1, and IL-10.^32–34^ Using the Transwell system, we further demonstrated that suppression of neutrophil effector functions is mediated by direct cell-to-cell interactions between stromal cells and neutrophils. Our observations are in agreement with a number of previous studies,^35–37^ including one in a model of corneal inflammation^35^ showing a direct interaction between keratocytes (a type of stromal cells) and neutrophils through ICAM1-CD18 binding.

Consistent with our in vitro observations, systemic treatment of mice with stromal cells results in significantly decreased expression of tissue-damaging factors, including MPO and the inflammatory cytokine IL-1β, by infiltrating neutrophils at the ocular surface. In addition, stromal cell–treated mice exhibited reduced ocular infiltration of neutrophils, which is consistent with previous reports showing that stromal cell–derived TSG-6 inhibits neutrophil migration to inflamed tissues.^10^ Moreover, this stromal cell–mediated suppression of neutrophil activation is accompanied by reduced ocular inflammation and a faster normalization of corneal tissue structure.

The current study elucidates the novel function of mesenchymal stromal cells in regulating neutrophil effector functions and limiting tissue damage in ocular inflammation. Our study provides new insights that may be utilized in the development of stromal cell–based therapeutic strategies for the prevention and treatment of ocular and nonocular tissue damage caused by excessive neutrophil activation.
